# A comparison of Selective Aortic Arch Perfusion and Resuscitative Endovascular Balloon Occlusion of the Aorta for the management of hemorrhage-induced traumatic cardiac arrest: A translational model in large swine

**DOI:** 10.1371/journal.pmed.1002349

**Published:** 2017-07-25

**Authors:** Ed B. G. Barnard, James E. Manning, Jason E. Smith, Jason M. Rall, Jennifer M. Cox, James D. Ross

**Affiliations:** 1 Air Force Trauma and Resuscitation Research Program, Joint Base San Antonio-Lackland, Texas, United States of America; 2 Institute of Naval Medicine, Alverstoke, United Kingdom; 3 Academic Department of Military Emergency Medicine, Royal Centre for Defence Medicine (Research & Academia), Medical Directorate, Birmingham, United Kingdom; 4 Department of Emergency Medicine, University of North Carolina at Chapel Hill, Chapel Hill, North Carolina, United States of America; 5 Emergency Department, Derriford Hospital, Plymouth, United Kingdom; 6 Division of Trauma, Critical Care & Acute Care Surgery, Oregon Health & Science University, Portland, Oregon, United States of America; Barts and the London School of Medicine & Dentistry Queen Mary University of London, UNITED KINGDOM

## Abstract

**Background:**

Survival rates remain low after hemorrhage-induced traumatic cardiac arrest (TCA). Noncompressible torso hemorrhage (NCTH) is a major cause of potentially survivable trauma death. Resuscitative Endovascular Balloon Occlusion of the Aorta (REBOA) at the thoracic aorta (Zone 1) can limit subdiaphragmatic blood loss and allow for IV fluid resuscitation when intrinsic cardiac activity is still present. Selective Aortic Arch Perfusion (SAAP) combines thoracic aortic balloon hemorrhage control with intra-aortic oxygenated perfusion to achieve return of spontaneous circulation (ROSC) when cardiac arrest has occurred.

**Methods and findings:**

Male Yorkshire Landrace cross swine (80.0 ± 6.0 kg) underwent anesthesia, instrumentation for monitoring, and splenectomy. TCA was induced by laparoscopic liver lobe resection combined with arterial catheter blood withdrawal to achieve a sustained systolic blood pressure <10 mmHg, cardiac arrest. After 3 min of arrest, swine were allocated to one of three interventions: (1) REBOA plus 4 units of IV fresh whole blood (FWB), (2) SAAP with oxygenated lactated Ringer’s (LR), 1,600 mL/2 min, or (3) SAAP with oxygenated FWB 1,600 mL/2 min. Primary endpoint was survival to the end of 60 min of resuscitation, a simulated prehospital phase. Thirty animals were allocated to 3 groups (10 per group)—5 protocol exclusions resulted in a total of 35 animals being used. Baseline measurements and time to cardiac arrest were not different amongst groups. ROSC was achieved in 0/10 (0%, 95% CI 0.00–30.9) REBOA, 6/10 (60%, 95% CI 26.2–87.8) SAAP-LR and 10/10 (100%, 95% CI 69.2–100.0) SAAP-FWB animals, *p* < 0.001. Survival to end of simulated 60-minute prehospital resuscitation was 0/10 (0%, 95% CI 0.00–30.9) for REBOA, 1/10 (10%, 95% CI 0.25–44.5) for SAAP-LR and 9/10 (90%, 95% CI 55.5–99.7) for SAAP-FWB, *p* < 0.001. Total FWB infusion volume was similar for REBOA (2,452 ± 0 mL) and SAAP-FWB (2,250 ± 594 mL). This study was undertaken in laboratory conditions, and as such may have practical limitations when applied clinically. Cardiac arrest in this study was defined by intra-aortic pressure monitoring that is not feasible in clinical practice, and as such limits the generalizability of findings. Clinical trials are needed to determine if the beneficial effects of SAAP-FWB observed in this laboratory study will translate into improved survival in clinical practice.

**Conclusions:**

SAAP conferred a superior short-term survival over REBOA in this large animal model of hemorrhage-induced traumatic cardiac arrest with NCTH. SAAP using an oxygen-carrying perfusate was more effective in this study than non-oxygen carrying solutions in TCA. SAAP can effect ROSC from hemorrhage-induced electrocardiographic asystole in large swine.

## Abstract 2

James Ross and colleagues use a laboratory model to evaluate aortic balloon catheter techniques for reversing trauma-induced cardiac arrest.

## Author summary

**Why was this study done?:**

- In the United States and the United Kingdom, trauma is the leading cause of death between 1 and 35 y of age, and the prospects for survival from cardiac arrest caused by trauma with bleeding that cannot be controlled by external compression is very low.
- Resuscitative Endovascular Balloon Occlusion of the Aorta (REBOA) uses an inflated catheter balloon in the largest artery of the body, the aorta, to limit blood loss from injured areas in the abdomen and pelvis to improve the chances of survival, but REBOA is most effective before the heart stops beating (cardiac arrest).
- Selective Aortic Arch Perfusion (SAAP) is an aortic balloon catheter resuscitation technique that provides infusion of oxygenated blood through the catheter to the heart during cardiac arrest with the aim of reversing cardiac arrest and improving the chances of survival.
- Aortic balloon catheter techniques, such as REBOA and SAAP, could potentially improve survival from trauma with uncontrolled bleeding, but they have not been directly compared when blood loss has resulted in cardiac arrest.
- Our study was designed to compare REBOA and SAAP in a laboratory model of trauma-induced cardiac arrest to evaluate their relative effectiveness for reversing cardiac arrest.

**What did the researchers do and find?:**

- We performed laboratory experiments in a swine model of traumatic liver injury with severe, uncontrolled bleeding that consistently resulted in rapid deterioration to cardiac arrest.
- We compared the success rate for reversing cardiac arrest, and surviving the subsequent hour, for the following 3 treatment regimens: (1) REBOA and intravenous blood transfusion, (2) SAAP with aortic infusion of lactated Ringer’s, a balanced salt solution, exposed to oxygen, and (3) SAAP with aortic infusion of fresh whole blood exposed to oxygen.
- We found that SAAP with oxygenated fresh whole blood was the most effective of the 3 treatments tested for both reversing cardiac arrest and surviving the subsequent hour.

**What do these findings mean?:**

- In this laboratory model, SAAP is a more effective resuscitation therapy than REBOA when severe, uncontrolled blood loss from abdominal trauma has resulted in cardiac arrest.
- SAAP with oxygenated fresh whole blood is more effective than SAAP with lactated Ringer’s solution.

## Introduction

More than 5 million people die each year as a result of injury—responsible for more deaths per year globally than tuberculosis, HIV, and malaria combined. [1] In the United Kingdom and the United States, trauma is the leading cause of death in those aged between 1 and 35 y old, [2,3] and as such is responsible for the greatest number of life years lost. Hemorrhage accounts for between 15% and 40% of trauma deaths. [4–8] Exsanguination from extremity hemorrhage is preventable—US military data has demonstrated an 85% reduction in death from extremity hemorrhage with the use of arterial limb tourniquets. [9] However, bleeding within the chest, abdomen, and pelvis (noncompressible torso hemorrhage—NCTH) is more difficult to control prehospital, and accounts for up to two-thirds of in-hospital hemorrhage deaths in the civilian setting. [6] UK military data, that includes prehospital deaths, provides a more accurate estimate—an 86% mortality in patients with NCTH; furthermore 88% of these patients died prehospital. [10]

Resuscitative Endovascular Balloon Occlusion of the Aorta (REBOA) utilizes a percutaneously deployed intravascular balloon catheter to effect the temporary occlusion of the aorta. [11–13] REBOA has demonstrated potential to manage NCTH in large animal models, [14–16] and clinical series, [17,18] and has been effectively used in the prehospital setting. [19] However, more recent publication of a large, retrospective, propensity score matched data study from Japan has shown an increased mortality with the use of REBOA. [20] In this series REBOA may have been used in a “last ditch” attempt at resuscitation. [20] This raises a question about the severity of hemorrhage at which endovascular occlusion of the aorta (REBOA) can be effective.

**Fig 1:**
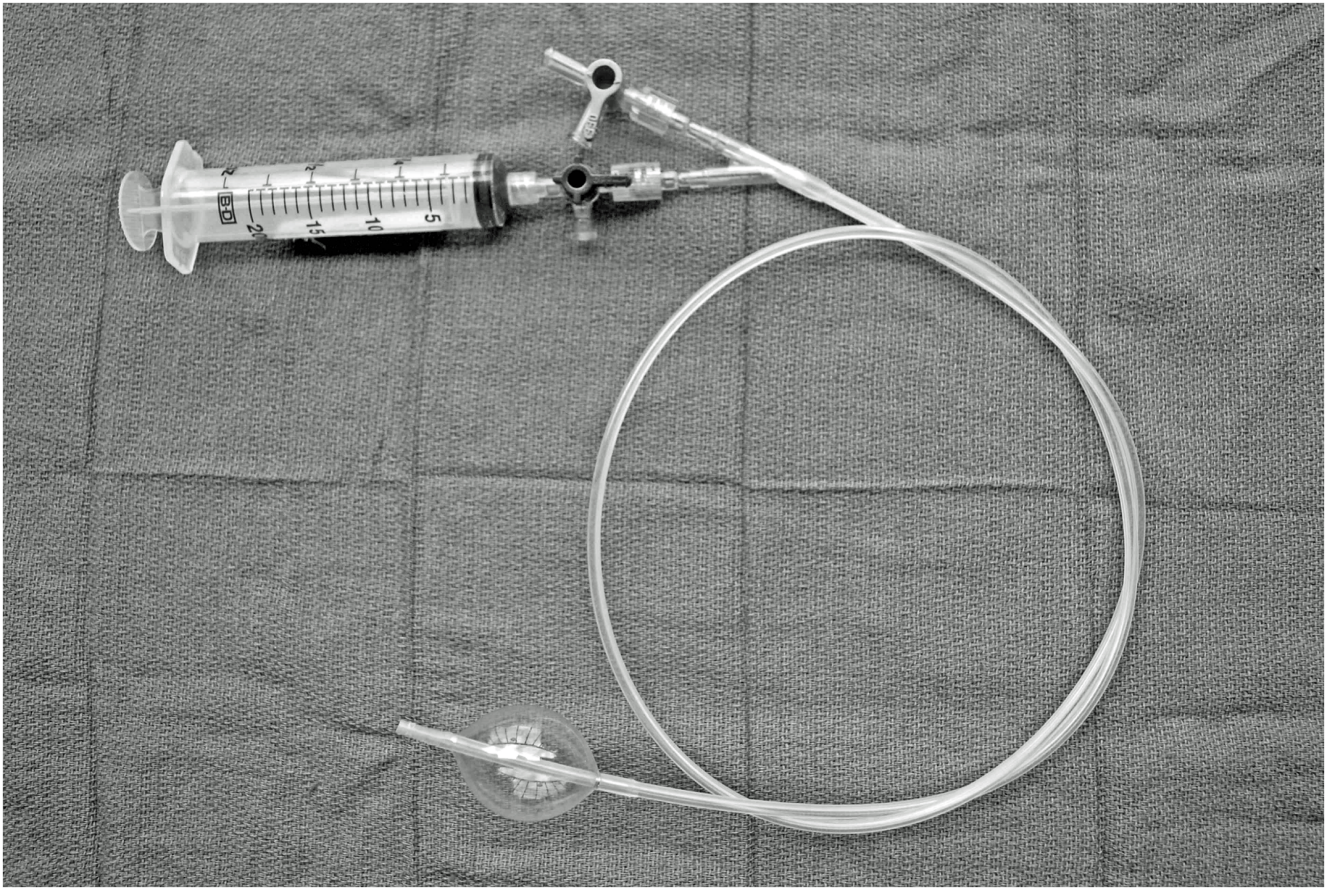
SAAP catheter (photograph by EGB Barnard). To determine the efficacy of SAAP in resuscitating NCTH-induced TCA compared to REBOA, we modified an existing large swine translational model of NCTH. [16] We hypothesized that in this translational model of NCTH-induced TCA, SAAP would significantly increase ROSC and 60-min survival (a surrogate for prehospital survival) as compared to REBOA, and that SAAP efficacy requires oxygen carrying capacity in the resuscitation fluid infused. NCTH, noncompressible torso hemorrhage; REBOA, Resuscitative Endovascular Balloon Occlusion of the Aorta; ROSC, return of spontaneous circulation; SAAP, Selective Aortic Arch Perfusion; TCA, traumatic cardiac arrest.

Selective Aortic Arch Perfusion (SAAP) is an experimental resuscitation technique that utilizes an endovascular balloon catheter to provide both aortic occlusion and intra-aortic infusion of oxygenated fluid cephalad to the level of the balloon, Fig 1. [21] In large animal models, SAAP has been shown to improve survival over conventional management in medical cardiac arrest, [22] and to be effective in managing hemorrhage-induced traumatic cardiac arrest (TCA) secondary to NCTH. [23]

## Methods

The experimental protocol was approved by the 59th Medical Wing Institutional Animal Care and Use Committee (IACUC). Experiments were performed at the 59th Medical Wing, United States Air Force, Office of the Chief Scientist, in a facility accredited by the American Association for the Accreditation of Laboratory Animal Care, and was conducted in accordance with guidelines established by the Public Health Service Policy on Humane Care and Use of Laboratory Animals and Office of Laboratory Animal Welfare. In keeping with international convention we applied the “3 Rs” principle of refinement, reduction, and replacement to this protocol.

### Refinement

Before any manipulation, all animals received a general anesthetic, delivered by a laboratory technician with specific experience and training in anesthesia of this species (under the direction of the Laboratory Animal Medical Officer). The level of anesthesia was continuously monitored using multiple parameters (including—mean alveolar concentration of inhaled anesthetic, heart rate, jaw muscle tone, palpebral reflexes, and graded pain response). The IACUC specifically approved this protocol as a non-survival study.

### Reduction

Comprehensive literature searches were undertaken to ensure that the hypothesis was both valid and not previously answered. We used the smallest number of animals that would provide meaningful results, and the IACUC biostatistician confirmed these calculations. Interim analysis was undertaken at *n* = 6 per group, to ensure that the hypothesis could not be answered using fewer animals.

### Replacement

We researched the use of mathematical models and in silico modeling, but the IACUC agreed that there was no acceptable replacement for live animals to answer the hypothesis.

### Protocol development

**Fig 2:**
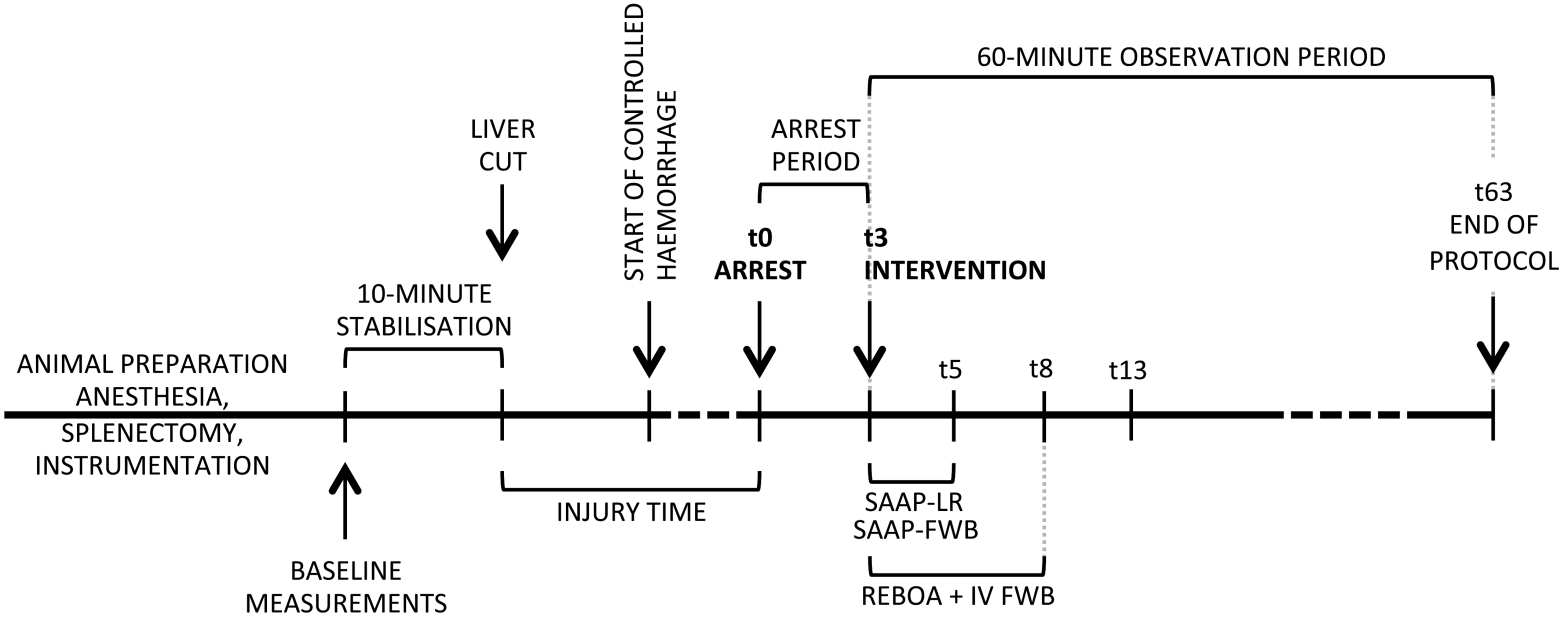
Protocol timeline. SAAP (inflation of an aortic occlusion balloon, and a 2-min intra-aortic oxygenated infusion of either LR solution, or FWB), REBOA (inflation of an aortic occlusion balloon, and a 5-min IV infusion of FWB). FWB, fresh whole blood; IV, intravenous; LR, lactated Ringer’s; REBOA, Resuscitative Emergency Balloon Occlusion of the Aorta; SAAP, Selective Aortic Arch Perfusion.

The model employed in this study was developed from a previously published protocol in our laboratory that describes the use of a laparoscopic liver injury to produce an uncontrolled NCTH. [24] The original model produced a mean systolic blood pressure (SBP) of 36 mmHg after injury, and was used to demonstrate efficacy of REBOA. [24] In order to evaluate the experimental intervention in a more severe state of hemorrhage, we added a controlled arterial bleed to the model that was executed in 4 stages: 1) anesthesia and instrumentation, 2) surgical preparation, 3) induction of hemorrhage-induced TCA by a hybrid of NCTH and controlled arterial hemorrhage, and 4) experimental intervention and 60 min of observation, Fig 2. The observation period was chosen based upon the best available data on the median prehospital time (61 min) observed in clinical practice of NCTH, [25] and that used in a previous large animal model of NCTH (60 minutes). [16]

### Blood transfusion

Whole blood was collected from a dedicated pool of animals between 24 and 48 h before experimentation. Each animal had up to 3 units (428.5 ml per unit) removed via the external jugular vein, and stored in standard citrated (CPDA-1) blood donation bags (TERUFLEX, Terumo Corp. Tokyo, Japan)—increasing the total volume per unit to 613 ml. Preinfusion samples of all infusates was undertaken to check hemoglobin, potassium and lactate concentrations, and oxygenation to confirm uniformity.

### Animal preparation

#### Animals

Male Yorkshire Landrace cross swine (*Sus scrofa*, 70–90 kg) were obtained from a single source animal vendor (John Albert, Cibolo, USA, 74-A-1246). Animals were quarantined for 72 h, and fasted for 18 h before experimentation with free access to water; experimental protocols began at 0630 hours. Thirty swine were allocated into 1 of 3 groups (randomized to 1 of 2 SAAP groups and a separate REBOA group), and investigators were blinded to randomization during the preparatory phases of the experiment. In the absence of prior experimental data to estimate effect size, the sample size was calculated to be capable of demonstrating a 40% delta in survival (primary outcome).

#### Anesthesia and instrumentation

Intramuscular ketamine (100 mg + 10 mg/kg), and 2 mg intramuscular buprenorphine were administered for initial sedation and analgesia. Animals were intubated via direct laryngoscopy with a cuffed endotracheal tube. Anesthesia was maintained on inhaled isoflurane (Abbott, North Chicago, IL, USA) between 1.5% and 2.5% throughout the protocol until hemorrhage-induced TCA, and recommenced in the same range if a return of spontaneous circulation (ROSC) was achieved. From intubation and induction of inhaled anesthesia to the initiation of the injury, end-tidal carbon dioxide (ETCO_2_) was maintained between 38 and 42 mmHg by alteration of ventilation rate—tidal volumes were fixed at 7 ml/kg. After baseline measurements, ventilator settings were not altered, allowing ETCO_2_ to be used as a surrogate for cardiac output (CO). [26] After induction of anesthesia the fraction of inspired oxygen was maintained at 0.35 until the start of the 10-min stabilization period immediately prior to the start of the injury, at which point it was reduced to 0.21 (equivalent to atmospheric air). At the start of the treatment intervention the inspired oxygen fraction was increased to 1.0 in order to emulate clinical management, Fig 2.

When anesthetized, electrocardiography, physiological monitoring, blood sampling, and vascular access lines were inserted percutaneously under ultrasound guidance. For physiological monitoring the following 8.5 Fr catheter introducers (Teleflex, Morrisville, NC, USA) were inserted: 1) Right carotid artery—intra-aortic arch blood pressure (BP) monitoring via a micromanometer-tipped catheter (Millar Inc. Houston, TX, USA), 2) Right external jugular vein (Swan-Ganz thermodilution catheter [Edwards Lifesciences, Irvine, CA, USA]), 3) Left femoral artery (invasive arterial pressure monitoring). An 8.5 Fr catheter was inserted into the left external jugular vein for blood infusion. A 5 Fr catheter was inserted into the left brachial artery for the collection of arterial blood samples. A 14 Fr vascular sheath (Cook Medical Inc. Bloomington, IN, USA) was inserted into the right common femoral artery to allow the controlled arterial hemorrhage, and subsequently to allow the passage of an intra-aortic balloon catheter. A midline cut-down to the left carotid artery was undertaken to place a flow probe (Transonic Systems Inc. Ithaca, NY, USA). Near-infrared spectroscopy (NIRS) tissue oxygenation monitoring pads (StO_2_—Terumo Corp. Tokyo, Japan) was placed on the skin overlying the right pectoralis muscle and the left medial thigh muscle.

#### Surgical preparation

Following placement of vascular lines and physiological monitoring, a splenectomy was performed via midline laparotomy. The *sus scrofa* spleen is contractile and theoretically provides an autotransfusion following injury that may affect the translation of results to clinical practice. The left lateral lobe of the liver was marked approximately 3–4 cm from the hilum with electrocautery in order to guide subsequent transection (to effect the NCTH). A direct cystostomy was performed, in order to prevent the physiological effects of a distended urinary bladder during the protocol. The spleen was returned to the abdominal cavity in order to restore normal abdominal volume, which could influence the volume of intra-abdominal hemorrhage. To guide the subsequent liver injury, 4 laparoscopic ports were inserted into the anterior abdominal wall. The peritoneum and anterior abdominal wall were then closed with a 1/0 vicryl continuous suture. Baseline physiological measurements and blood samples were then obtained, followed by a 10-min stabilization period, Fig 2.

#### NCTH injury and controlled arterial hemorrhage

At the end of the 10-min stabilization period, the abdomen was insufflated via a laparoscopic port to a pressure of 12 mmHg. The left lateral lobe of the liver was visualized, and approximately 70% excised with 5 mm Metzenbaum endoshears. At completion of the liver transection, the abdomen was rapidly desufflated, instruments and ports removed, and the laparoscopic holes approximated with surgical skin staples. After 5 min of liver free-bleeding, a controlled hemorrhage was initiated at 3 ml/kg/min from the left femoral artery. At the time of arrest (onset of TCA − t0, SBP <10 mmHg) the controlled hemorrhage was continued at 1 ml/kg/min, for up to 2 min, if the intra-aortic SBP >0 mmHg, Fig 2.

#### Intervention

Three minutes after the onset of hemorrhage-induced TCA (t3), animals were allocated to 1 of 3 groups: a) REBOA with 4 units of IV fresh whole blood (FWB), b) SAAP with 1,600 ml of intra-aortic oxygenated lactated Ringer’s solution (SAAP-LR), or c) SAAP with 1,600 ml of intra-aortic oxygenated fresh whole blood (SAAP-FWB). A web-based computer software program (randomizer.org) was used for randomization of the SAAP groups. In all 3 groups, an intra-aortic balloon catheter (11.5 Fr Outer Diameter, 7.5 Fr Inner Diameter, 80 cm working length, 30 mm outer balloon diameter with 17 ml fluid inflation; Vention Medical, Inc., New Jersey, USA) was advanced above the diaphragm into aortic Zone 1 under fluoroscopy, and inflated.

a) REBOA Group–The balloon catheter lumen was flushed with LR solution prior to insertion and then clamped with a 3-way stopcock for the duration of the protocol. Immediately following balloon catheter inflation, warmed FWB was infused via the left external jugular vein catheter at a rate of 500 ml/min using a Belmont rapid infuser (Belmont Instrument Corporation, Billerica, MA, USA). The total volume of 4 units of blood, 2,452 ml, was infused over 5 min. In order to compensate for citrate chelation of calcium in the stored blood product, and thereby prevent systemic ionized hypocalcaemia, an IV infusion of 10% calcium chloride was administered at a rate of 6.94 ml/min. The required dose of calcium chloride was previously confirmed in both ex vivo and in vivo experiments. No further fluid boluses were given.

b) SAAP-LR Group–Immediately following balloon inflation, warmed oxygenated intra-aortic LR solution was infused at a rate of 800 ml/min (approximately 10 ml/kg/min) for 2 min (total infusion volume = 1,600 ml). If following the initial SAAP-LR infusion, the SBP was <90 mmHg, then up to 7 250-ml boluses of oxygenated intra-aortic LR solution were given.

c) SAAP-FWB Group–Immediately following balloon inflation, warmed oxygenated FWB was infused at a rate of 800 ml/min (approximately 10 ml/kg/min) for 2 min (a total infusion volume of 1,600 ml). In order to compensate for citrate chelation of calcium in the stored blood product, and thereby prevent systemic ionized hypocalcaemia, 10% calcium chloride was added to the SAAP infusion, immediately prior to entry into the animal, at a rate of 11.1 ml/min. The required dose of calcium chloride was previously confirmed in both ex vivo and in vivo experiments. If following the initial SAAP-FWB infusion the SBP was <90 mmHg, then up to 7 250-ml boluses of oxygenated intra-aortic FWB were given, together with a balanced 10% calcium chloride infusion as previously described.

### SAAP circuit

**Fig 3:**
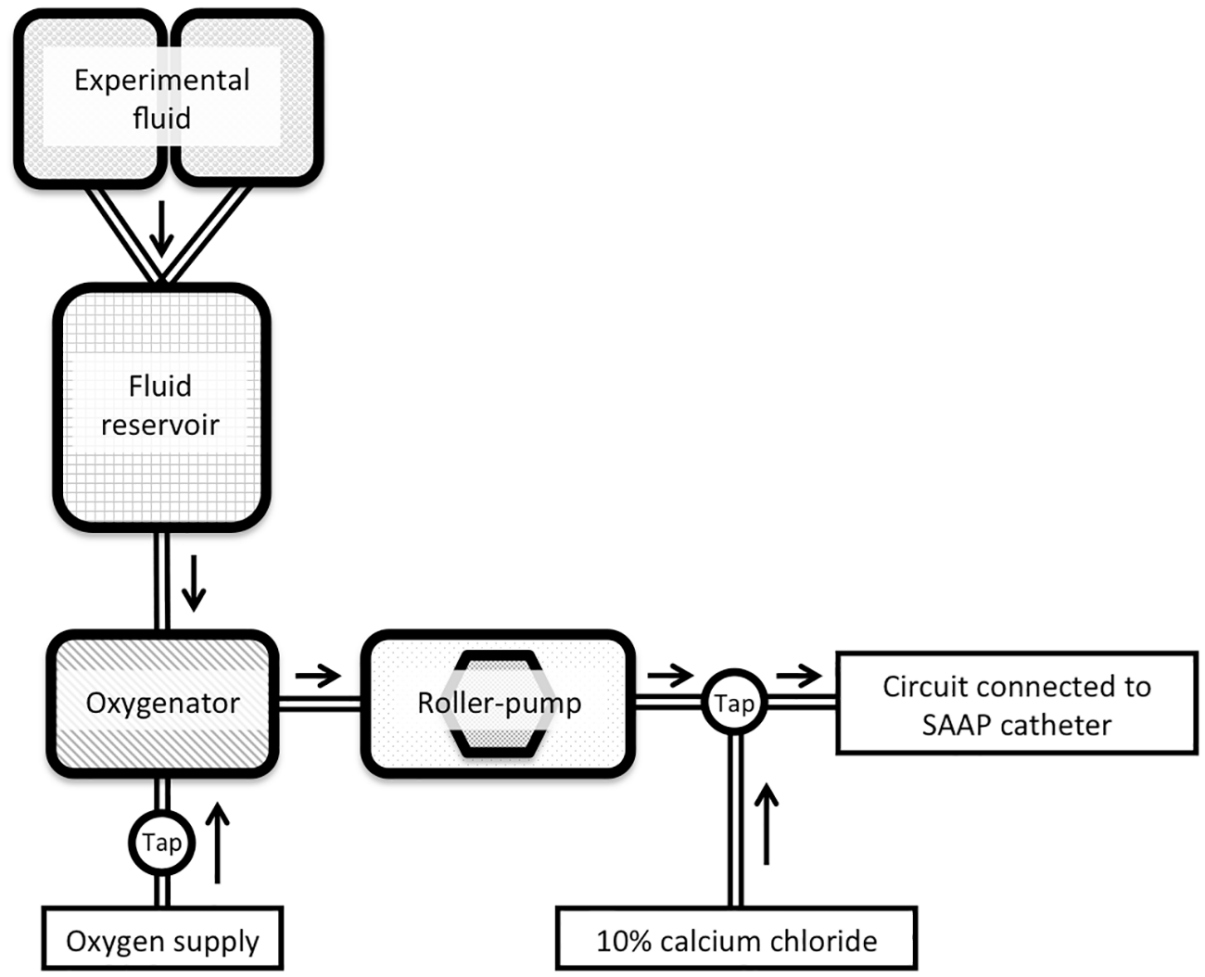
SAAP circuit. SAAP, Selective Aortic Arch Perfusion.

Bespoke SAAP circuits were constructed, and consisted of a reservoir (Belmont Rapid Infuser reservoir– 3L), an oxygenator (Quadrox, Maquet IND, Wayne, NJ, USA), tubing (3/8” ID ECMO circuit, Maquet INC, Wayne, NJ, USA), and Masterflex peristaltic pumps, Fig 3. This apparatus included ports for recirculation during preparation, infusate sampling, 10% calcium chloride infusion, and to allow a rapid bolus of 50 ml of LR solution to be given immediately prior to the start of the SAAP infusion. This 50-ml bolus has previously been demonstrated under fluoroscopy to close the aortic valve, thereby allowing maximal flow into the coronary vessels by preventing retrograde filling of the left ventricle.

### Protocol definitions and end-points

TCA (t0) was defined as SBP < 10 mmHg, together with an electrocardiographic heart rate lower than preinjury baseline. ROSC was defined as an SBP ≥ 50 mmHg 10 min after the start of the intervention (t13) (in keeping with the assumption that, clinically, a central pulse would not be palpable < 50 mmHg). [27] “Death in protocol” was defined as an SBP < 50 mmHg sustained for 2 min. Animals without an ROSC at t13 were observed for a further 2 min, and if they met “death in protocol” criteria, no further data observations were recorded. Additionally, in animals that had an initial ROSC, but subsequently met “death in protocol” criteria, no further data observations were recorded. This clinically-relevant method allows recognition of unsuccessful resuscitation from TCA (death), and also the ability to report the central tendency of physiological variables in animals that are effectively resuscitated (survive). Survival was defined as an SBP ≥ 50 mmHg 60 min after the start of the intervention (t63), Fig 2.

### Postexperimental procedures

At the end of the protocol, animals were euthanized by a trained laboratory technician with intravenous (IV) administration of 100 mg/kg of sodium pentobarbital (Euthanasia-5 solution), in accordance with the American Veterinary Medical Association euthanasia guideline. Following animal euthanasia, the liver was removed in order to quantify the laparoscopic liver injury as a percentage-by-weight of the left lateral lobe excised. The free intraperitoneal blood and clots (hemoperitoneum) were weighed to quantify the NCTH. The volume of controlled arterial hemorrhage was measured within 10 min of collection.

### Data acquisition

A data acquisition system was used to record data points at 500 Hz of intra-aortic arch BP and carotid blood flow—these data were analyzed as mean values every 5 sec. Other variables were recorded, or automatically calculated, at 60-sec intervals (heart rate, ETCO_2_, central venous pressure [CVP], systemic vascular resistance [SVR], CO, stroke volume [SV], and core temperature). NIRS values were recorded 11.55 times per min, as dictated by the manufacturer’s software (and are reported as a percentage of the baseline value).

Blood samples obtained from the left brachial artery were collected at baseline, time of arrest, 10 min after intervention, and subsequently at 10-min intervals until the end of the protocol. Variables recorded included: arterial blood gas sample (pH, pO_2_, pCO_2_, K^+^, ionized Ca^++^, lactate, base deficit), hematology sample (hemoglobin, platelet count), clotting profile sample (prothrombin time, and thromboelastography [R, K, alpha angle, and maximum amplitude]).

#### Primary outcome

60-min survival postintervention (as a surrogate for prehospital survival).

#### Secondary outcomes

ROSC (defined as a SBP ≥ 50 mmHg 10 min after the intervention). In animals with an ROSC: physiological monitoring (SBP, left carotid artery flow, ETCO_2_, and NIRS), and arterial blood gas samples (hemoglobin, pH, lactate, base deficit, K^+^, ionized Ca^++^, and clotting profile, including thromboelastography) at the end of the protocol (60 min after the intervention).

### Data analysis

Data are reported as means (± standard deviation); 60-min survival and ROSC at 10 min as percentages (95% confidence intervals). Baseline characteristics were analyzed by one-way analysis of variance. Within-group comparisons were made using Student *t* test (two-tailed). Statistical analysis of 60-min survival was by Log-rank (Mantel-Cox) analysis, and analysis of ROSC percentage between groups was with Fisher’s exact test (GraphPad Prism, La Jolla, CA, USA). Statistical significance was predefined as *p* < 0.05.

## Results

### Baseline characteristics

**Fig 4:**
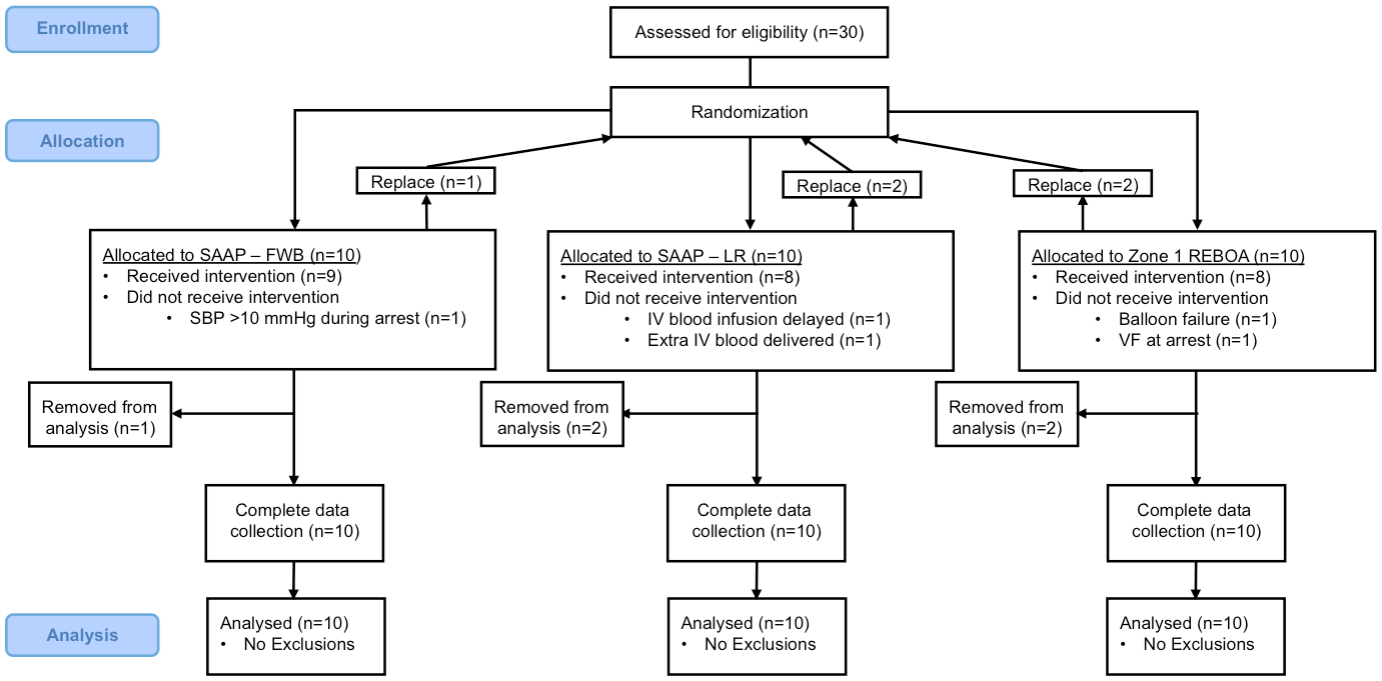
Consort diagram.

Thirty animals were allocated to 1 of 3 groups (*n* = 10 per group), Fig 4. Five experimental animals were excluded owing to predefined protocol exclusion criteria, and replacements were subsequently added to the schedule—therefore, a total of 35 animals were used for this study. Exclusions: REBOA group (2)– 1 received IV blood infusion 30 sec later than protocol dictated and 1 erroneously received further IV blood volume after the initial infusion; SAAP-LR group (1)– 1 had an SBP throughout the arrest period >10 mmHg; SAAP-FWB group (2)– 1 experienced SAAP catheter balloon failure and 1 entered ventricular fibrillation during the arrest period.

Baseline measurements, obtained immediately prior to the start of the injury, were consistent between groups and can be found in Table 1.

**Table 1:** Animal baseline and injury characteristics.

Baseline measurements	REBOA Mean (±SD) *n* = 10	SAAP-LR Mean (±SD) *n* = 10	SAAP-FWB Mean (±SD) *n* = 10	ANOVA *p*-value
Heart rate (beats/min)	89.8 (±15.7)	89.9 (±14.9)	86.4 (±11.8)	*p* = 0.82
SBP (mmHg)	86.3 (±5.8)	81.7 (±5.4)	88.3 (±9.0)	*p* = 0.11
CO (l/min)	5.8 (±1.2)	6.0 (±1.4)	5.8 (±0.77)	*p* = 0.77
Left carotid artery flow (ml/min)	395 (±91)	360 (±86)	359 (±55)	*p* = 0.52
CVP (mmHg)	9.9 (±1.3)	9.4 (±2.6)	9.9 (±1.5)	*p* = 0.79
**Injury characteristics**				
Animal weight (kg)	82.5 (±4.4)	77.6 (±5.9)	79.8 (±6.5)	*p* = 0.17
Spleen weight (g)	612 (±171)	623 (±225)	669 (±207)	*p* = 0.80
Injury time (sec)	614 (±139)	719 (±94)	700 (±71.8)	*p* = 0.08
Proportion of liver lobe excised (%)	69.0 (±5.8)	70.5 (±10.8)	67.8 (±10.5)	*p* = 0.81
Total hemorrhage (ml/kg)	42.8 (±6.7)	44.2 (±5.1)	37.5 (±5.5)	*p* = 0.04*

CO, cardiac output; CVP, central venous pressure; REBOA, Resuscitative Endovascular Balloon Occlusion of the Aorta; SAAP-FWB, Selective Aortic Arch perfusion with 1,600 ml of intra-aortic oxygenated fresh whole blood; SAAP-LR, Selective Aortic Arch Perfusion with 1,600 ml of intra-aortic oxygenated lactated Ringer’s solution; SBP, systolic blood pressure.

### Injury characteristics

The mean animal weight was 80.0 kg (± −6.0). The mean time from the start of the injury to the onset of hemorrhage-induced TCA (t0) was just under 11 min and was consistent between groups, as was the spleen weight, and the percentage-by-weight of the liver lobe excision. The total hemorrhage was significantly lower in the SAAP-FWB group, Table 1. The mean blood loss per animal (a combination of hemoperitoneum and controlled hemorrhage) was 3,318 ml (±552). This resulted in a severe model of TCA: immediately prior to the intervention, the mean intra-aortic SBP was 1.5 mmHg (±2.2), the left carotid artery flow was 0.2 ml/min (±1.1), the mean electrocardiographic heart rate was 44 min^−1^ (±32), and 9 of 30 (30%) animals were in electrocardiographic asystole.

### Intervention characteristics

The intervention (t3) was commenced a mean of 184 sec (±5.1) after the start of hemorrhage-induced TCA. The active oxygenation of the SAAP-FWB perfusate effected some significant differences in properties of this infusion compared to the REBOA group (pH 7.50 ± 0.12 versus 7.04 ± 0.02 and pO_2_ 621.6 ± 53.5 versus 61.4 ± 16.3, respectively, *p* = 0.001). In addition, the REBOA group’s perfusate had a significantly higher hemoglobin concentration but was not determined to be physiologically relevant to the intervention (7.9 ± 0.8 versus 7.0 ± 0.6, *p* = 0.01).

The total FWB volume infused for the REBOA group was 2,452 ml per animal (as per protocol). The SAAP-FWB group received a mean number of 2.6 (±2.4) 250-ml boluses after the initial treatment of 1,600 ml per animal. However, the total infusion volume of FWB was lower, though not statistically significant, in the SAAP-FWB group compared to the REBOA group (2,250 ml ± 594 versus 2,452 ml ± 0.0, respectively).

### Primary outcome

**Fig 5:**
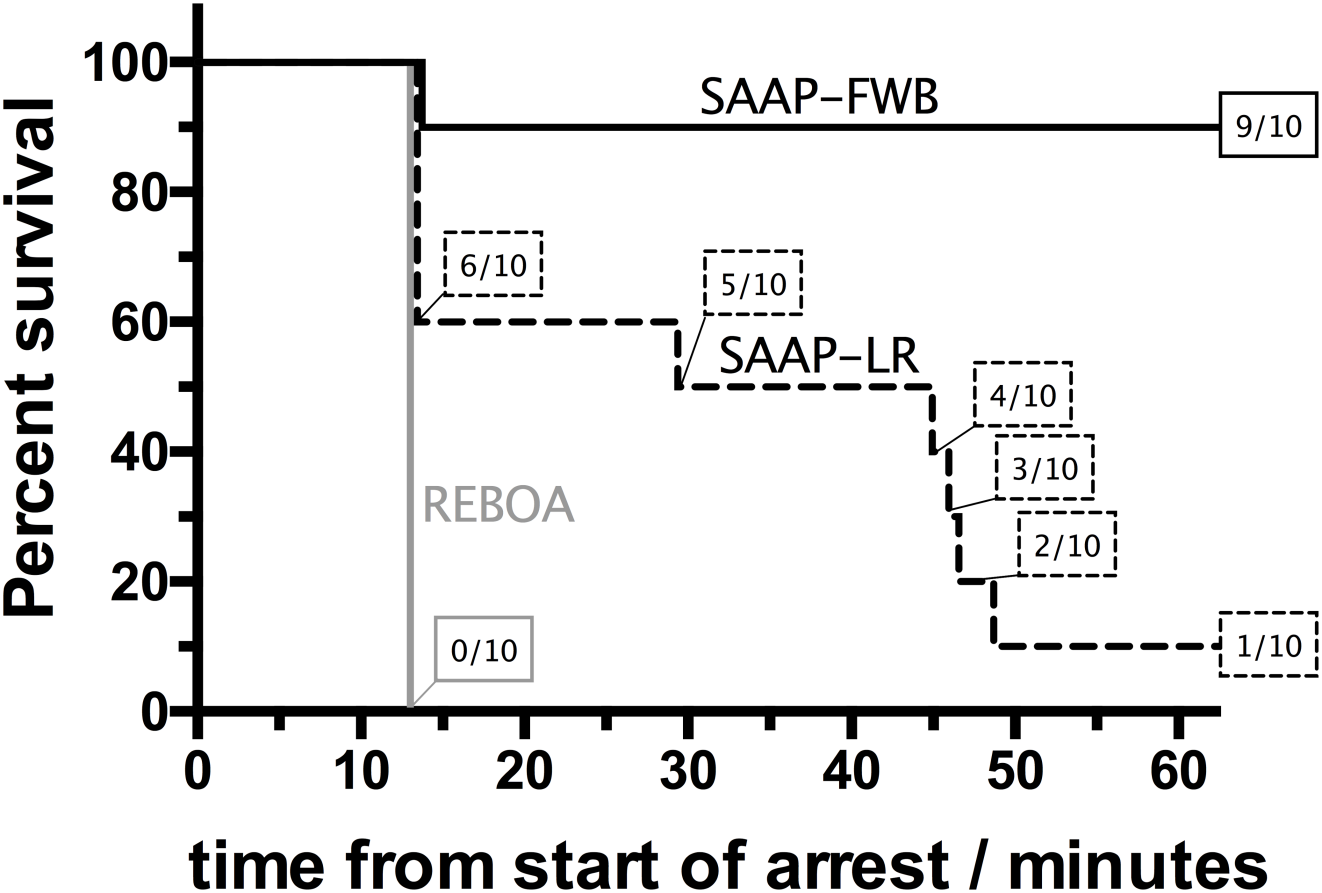
Primary outcome. Prehospital (60-min) survival of REBOA group (*n* = 10), compared to SAAP-LR group (*n* = 10), and SAAP-FWB group (*n* = 10). REBOA, Resuscitative Endovascular Balloon Occlusion of the Aorta; SAAP-FWB, Selective Aortic Arch perfusion with 1,600 ml of intra-aortic oxygenated fresh whole blood; SAAP-LR, Selective Aortic Arch Perfusion with 1,600 ml of intra-aortic oxygenated lactated Ringer’s solution.

None of the REBOA animals (*n* = 10) survived the 60-min observation period, 1 of 10 (10%) of the SAAP-LR animals and 9 of 10 (90%) of the SAAP-FWB animals survived, *p* < 0.001, Fig 5.

### Secondary outcomes

#### REBOA group

**Fig 6:**
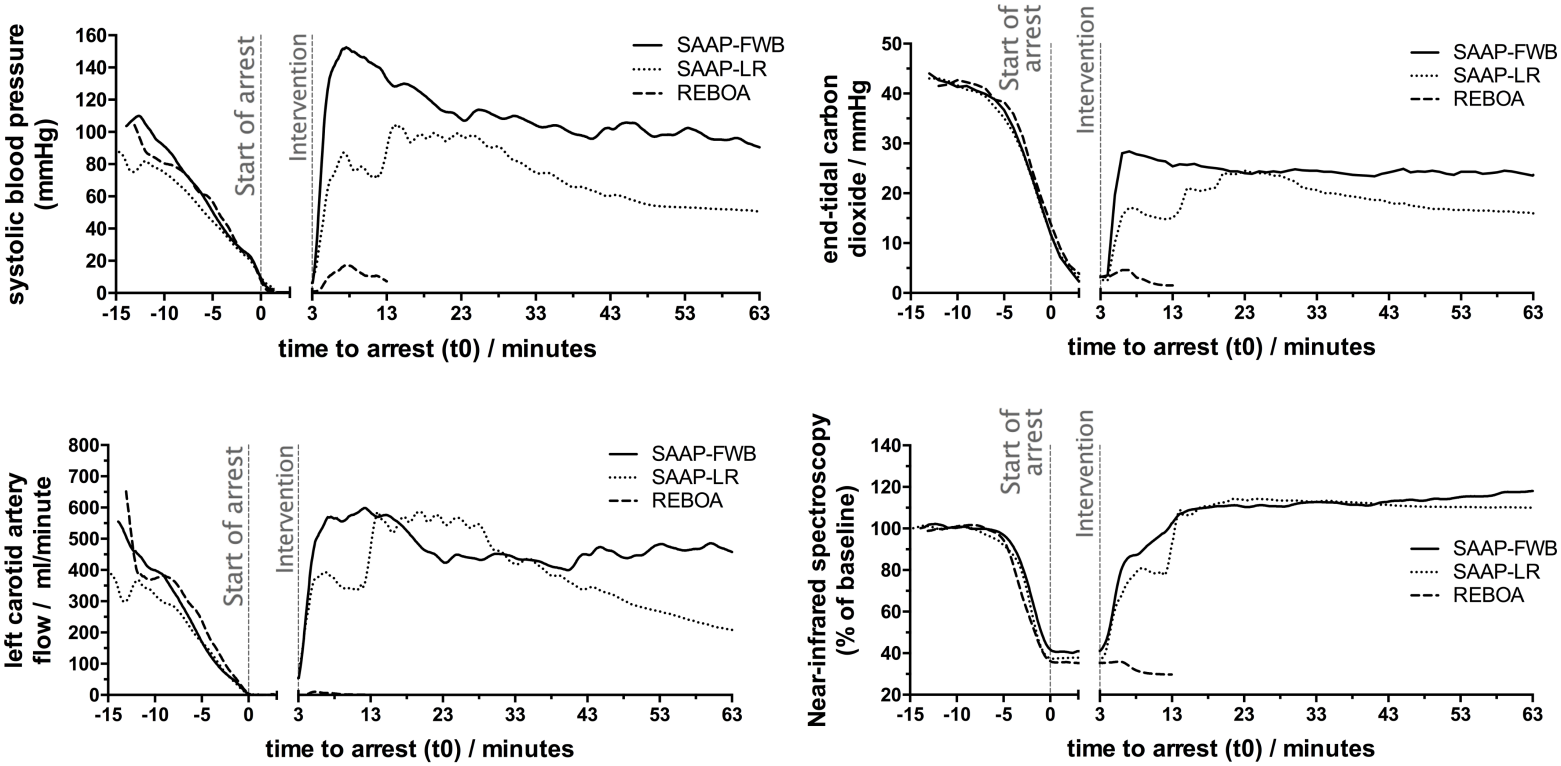
Secondary outcomes. Mean SBP, left carotid artery flow, ETCO_2_, and proximal NIRS throughout the protocol. ETCO_2_, end-tidal carbon dioxide; NIRS, near-infrared spectroscopy; SBP, systolic blood pressure.

There was a small increase in SBP in the REBOA group during the intervention, from a mean of 2.3 mmHg (±2.3) to a mean of 19.0 mmHg (±4.8), Fig 6. The diastolic BP was also observed to increase, from a mean of 0.8 mmHg (±1.2) to a mean of 16.4 mmHg (±5.1), but there was no carotid artery flow (Fig 6) and no increase in heart rate. None of the REBOA group had ROSC, and therefore all were dead 10 min after the start of the intervention (t13), Fig 6.

#### SAAP-LR group

Six of ten animals in the SAAP-LR group achieved ROSC but only 1 survived to t63. At t13, after LR-SAAP infusion, the mean hemoglobin was 3.0 g/dL (±2.0). There was also evidence of clotting dysfunction with a mean maximum clot amplitude on thromboelastography of 46.0 mm (±29) in the SAAP-LR group at this time, compared to 74.1 mm (±2.8) in the SAAP-FWB group.

#### SAAP-FWB group

All 10 animals of the SAAP-FWB group achieved ROSC. However, one animal had a rapidly falling BP, and met “death in protocol” criteria 13 min after the intervention (t16). Post-mortem analysis demonstrated a percentage-by-weight left lateral lobe of the liver excision that was 2 standard deviations higher than the mean of the other nine animals, 84.9% (281 of 331 grams) as compared to 65.9% (±9.4), respectively. The liver vascular anatomy was also noted to contain an abnormally wide caliber vein, and in essence the injury was a very proximal, unsurvivable, central venous hemorrhage.

The remaining 9 animals that survived the 60-min observation period required a mean of 528 ml (±492) in additional SAAP blood infusion to maintain SBP > 90mmHg. Four of nine (44%) of this group were in electrocardiographic asystole (or p-wave asystole) during the arrest period. However, no animals were observed to have arrhythmias during the observation period.

In order to characterize the effect of SAAP-FWB intervention, the baseline characteristics of the surviving animals were compared to the end of the 60-min observation period. The heart rate, SBP, left carotid artery flow, proximal NIRS, lactate and base deficit were all significantly higher at the end of the observation period compared to baseline. The hemoglobin concentration, platelet count, and prothrombin time were statistically lower at the end of the protocol. However, the clotting function measured by thromboelastography was statistically comparable, Table 2.

**Table 2:** SAAP-FWB group survivors (*n* = 9) comparison of baseline and end-of-protocol physiology.

**Physiology**	**Baseline** Mean (+/−SD)	**t63** Mean (+/−SD)	***t* test** *p*
Heart rate (beats/min)	85.6 (+/−12.1)	123 (+/−11.7)	<0.001*
SBP (mmHg)	87.9 (+/−9.4)	94.5 (+/-7.2)	0.04*
Mean arterial pressure (mmHg)	67.9 (+/−6.7)	77.0 (+/-3.2)	0.004*
CO (l/min)	5.6 (+/−0.6)	3.2 (+/−0.6)	<0.001*
SV (ml)	70.3 (+/−20.9)	24.4 (+/-5.5)	<0.001*
ETCO_2_ (mmHg)	39.8 (+/−1.3)	24.2 (+/−2.6)	<0.001*
SVR (dyne/sec)	949 (+/−203)	2041 (+/-485)	<0.001*
Left carotid artery flow (ml/min)	345 (+/−40.6)	491 (+/−74.5)	<0.001*
CVP (mmHg)	9.8 (+/−1.6)	6.7 (+/−1.9)	0.001*
Central temperature	38.0 (+/−0.6)	37.3 (+/−0.4)	0.003*
**Arterial blood gas sample**	**Baseline** Mean (+/−SD)	**t63** Mean (+/−SD)	***t* test** *p*
pH	7.47 (+/−0.03)	7.35 (+/−0.05)	<0.001*
pO_2_ (mmHg)	130 (+/−17.4)	431 (+/−33.9)	<0.001*
pCO_2_ (mmHg)	37.7 (+/−1.3)	25.6 (+/−2.5)	<0.001*
Potassium (mmol/l)	4.4 (+/−0.3)	5.4 (+/−0.5)	<0.001*
Ionized Calcium (mmol/l)	1.2 (+/−0.0)	1.3 (+/−0.2)	0.13
Lactate (mmol/l)	1.6 (+/−0.5)	14.2 (+/−1.2)	<0.001*
Base deficit	−5.9 (+/−2.0)	10.6 (+/−2.0)	<0.001*
**Hematology** **& thromboelastometry**	**Baseline** Mean (+/−SD)	**t63** Mean (+/−SD)	***t* test** *p*
Hemoglobin (g/dl)	10.9 (+/−1.1)	9.2 (+/−0.8)	<0.001*
Platelet count (x10^9^/l)	292 (+/−81.2)	242 (+/−47.9)	0.04*
Prothrombin time (sec)	14.2 (+/−0.4)	13.5 (+/−0.4)	<0.001*
R (sec)	4.3 (+/−0.4)	4.6 (+/−1.5)	0.64
K (sec)	1.2 (+/−0.3)	1.6 (+/−1.1)	0.29
Alpha-angle (°)	71.4 (+/−6.5)	66.5 (+/−8.4)	0.28
Maximum amplitude (mm)	76.6 (+/−4.7)	73.5 (+/−3.7)	0.16
**StO**_**2**_ (% of baseline value)	**Baseline** Mean (+/−SD)	**t63** Mean (+/−SD)	***t* test** *p*
Proximal (%)	100 (+/−0.0)	118 (+/−7.5)	<0.001
Distal (%)	100 (+/−0.0)	63.6 (+/−4.8)	<0.001

“*” indicates a statistically significant difference from baseline measure.

CO, cardiac output; ETCO_2_, end-tidal carbon dioxide; CVP, central venous pressure; SBP, systolic blood pressure; SV, stroke volume; SVR, systemic vascular resistance.

## Discussion

This study has demonstrated that SAAP-FWB infers a significant 60-min survival advantage over Zone 1 REBOA in this large swine translational model of hemorrhage-induced TCA. It has also shown that in large swine, SAAP can effect ROSC from electrocardiographic asystole.

This is a new finding and one that contradicts previously taught doctrine about survivability following TCA. Only a decade ago, hemorrhage-induced TCA was thought to be almost always fatal, [28] and historically, attempted resuscitation following TCA was deemed futile. [29] However, since then, both military and civilian case series have highlighted the potential survivability following hemorrhage-induced TCA. One recent study reports an overall 30-d survival rate of 7.5%, but for those with a predominant hemorrhagic injury pattern, this was 8.4%. [30] Recent studies examining functional outcomes have also produced positive results with between 63% and 90% of TCA survivors having a moderate to full neurological recovery. [30,31]

This positive shift in potential survivability of the most extreme form of hemorrhage is concurrent with the institution of hemostatic resuscitation treatment protocols and the early aggressive management of such patients with rapid infusion of blood and blood products. [32–34] However, this study has gone one step further and shown that even those with electrocardiographic asystole following TCA could potentially be salvageable with the endovascular perfusion support provided by SAAP resuscitation.

This study also challenges the previous guidance that termination of resuscitation decisions can reliably be based on the finding of absence of electrical activity, or asystole. [35] There have now been reported cases of successful resuscitation from asystole, [31,36] and with the technical capability evolving, it may be possible in the future to salvage a proportion of these patients.

All previous published large animal studies examining the efficacy of REBOA have used less severe models of hemorrhage. These studies have demonstrated that REBOA is superior to no treatment in critical hypovolemia and causes less physiological derangement than thoracotomy and aortic cross-clamping. [16,37,38] There is a single clinical report of successful REBOA deployment in a patient in hemorrhagic TCA. [18] This patient had an in-hospital pulseless electrical activity (PEA) arrest, and was successfully managed with Zone 1 REBOA and blood products. It is likely that this patient, although technically in TCA (no palpable pulse and an electrocardiographic heart rate of 130 min−^1^), was in fact in a low-output state in trauma (LOST). [30]

Traumatic hemorrhage is a spectrum of disease: at the less severe end of the spectrum, the patient has a low BP with a palpable pulse (critical hypovolemia), which is potentially reversible with rapid infusion of blood and blood products and definitive anatomical control of hemorrhage. If the hemorrhage is uncontrolled, this is followed by hemorrhage-induced TCA—hemorrhage without a palpable pulse but with ongoing CO (LOST). [30,34] It is likely that REBOA, together with IV fluid resuscitation, could be effective in managing both critical hypovolemia and LOST secondary to NCTH. However, with ongoing hemorrhage, LOST will progress into a no-output state in trauma (NOST), where there is no or extremely low CO. Control of ongoing hemorrhage (REBOA) and IV filling alone will therefore be ineffective in the majority of cases, as the heart is no longer capable of providing a circulation. This requires an increasing level of aggressive resuscitation in combination with technical advances such as SAAP.

A gap-analysis of 397 patients in England and Wales with anatomical and physiological indications for REBOA demonstrated that up to 40% were in TCA on arrival to hospital. [39] It is logical that earlier hemorrhage control may prevent TCA in patients with an injury pattern that is amenable to REBOA. However, prehospital use of REBOA is currently only delivered by a single urban physician-led team. [40] This reflects not only the technical challenge, but also the lack of strong evidence of benefit and clear guidance as to the indications for this potentially risky intervention. Patients in TCA present less of a treatment dilemma than those with critical hypovolemia. However, there is limited evidence that passive aortic occlusion (REBOA) is an effective intervention for TCA. Active endovascular resuscitation (for example SAAP) has been shown in a previous animal model of hemorrhage-TCA to be effective at a mean arterial pressure of <10 mmHg. [23]

Our study utilized a very severe model of TCA, thereby demonstrating that REBOA and rapid high-volume venous filling is ineffective at resuscitating those with no or very low CO. The small increase in both SBP and diastolic BP, in the absence of carotid artery flow or an increase in heart rate observed in this study is likely to be due to high-volume IV filling alone. SAAP with oxygenated FWB was demonstrated to be effective, with all animals having a ROSC. Although SAAP is more technically and logistically challenging than REBOA, prehospital extracorporeal life support (ECLS) for medical arrest in France in recent years demonstrates the feasibility of performing advanced, complex extracorporeal perfusion interventions outside the hospital setting. [41] ECLS requires arterial and venous cannulas that are larger than an SAAP catheter, together with a closed venoarterial perfusion circuit. SAAP requires only femoral arterial access without the need for a closed venoarterial circuit to initiate resuscitative perfusion and can be performed with lightweight, commercially-available perfusion and oxygenation devices that allow for portability into the prehospital setting.

One of the challenges to implementation of SAAP, particularly in the prehospital setting or military theater of operations, is the need for an oxygen-carrying perfusate. FWB served as the oxygen carrier in the present study and was shown to be highly effective. However, the availability of stored blood products outside of a hospital, either whole blood or blood products (pack red blood cells and fresh frozen plasma), is highly dependent upon cold chain logistic capabilities that are not readily available in many civilian or military settings. Previous laboratory studies have also utilized hemoglobin-based and perfluorocarbon-based oxygen carriers with similar success, but there are presently no widely available regulatory agency-approved hemoglobin or perfluorocarbon oxygen carriers. Many advanced prehospital care systems, including air ambulances and physician response units, now carry blood products routinely. South Africa has, for many years, had regulatory approval for use of hemoglobin-based oxygen carrier-201 (Hemopure, HbO2 Therapeutics, Inc., Cambridge, MA), a room-temperature stable product with a shelf-life of several years. Thus, the availability of blood products and other oxygen carriers does present a challenge, but not an insurmountable one.

REBOA appears to be best suited for patients with NCTH that still have an intact, albeit compromised, intrinsic circulation, whereas SAAP is a therapy designed primarily to treat cardiac arrest, a state of lost intrinsic circulation. However, the interface between these 2 states is a severely compromised circulation with impending cardiac arrest. This interface poses a challenge to clinically determine which patients would best benefit from REBOA versus SAAP. Heart rate, point-of-care cardiac ultrasound, and clinical assessment of compensatory reserve versus impending cardiac arrest could be used to guide the choice of catheterization for REBOA versus SAAP. Tachycardia suggests ongoing compensatory effort by the heart, whereas a rapidly dropping heart rate (inappropriate bradycardia) indicates impending cardiac arrest. Cardiac ultrasound could differentiate active contractility in the face of hypovolemia versus loss of contractility heralding arrest. Clinical assessment of the rate of decompensation could help differentiate patients who have time for REBOA catheterization and IV volume resuscitation versus patients who will likely progress to cardiac arrest during aortic catheterization and would thus be more appropriate for SAAP therapy. Future clinical experience with both REBOA and SAAP will reveal the best approach to this interface between these 2 interventions.

Whilst it is challenging to clinically determine which patients would benefit from active endovascular resuscitation as compared to passive endovascular resuscitation (REBOA), SAAP allows for a REBOA-like intervention (inflation of the SAAP balloon without aortic infusion) prior to full SAAP treatment. This may therefore present the potential of a clinical paradigm that begins with passive resuscitation, followed by active resuscitation (SAAP), as required. The viability for SAAP as a multirole catheter will be highly dependent on catheter size and simplicity of function when used as an afterload support and inflow control device only.

### Limitations

This study was undertaken under laboratory conditions in large swine, and as such may have practical limitations when applied in human clinical trials or general clinical practice. In particular, the current clinical definition of TCA does not differentiate between a LOST (where REBOA and IV filling may be effective) and a NOST (where SAAP is likely to be superior to REBOA and IV filling). There is some evidence from this study that cardiac electrical activity and ETCO_2_ may be of utility in making this differentiation. Furthermore, the complexity of delivering SAAP in-hospital, and particularly prehospital should not be underestimated.

### Conclusion

SAAP inferred a superior short-term survival over REBOA in this large animal model of hemorrhage-induced TCA with NCTH. SAAP using an oxygen-carrying perfusate is more effective than non-oxygen carrying solutions in TCA. SAAP can effect ROSC from hemorrhage-induced electrocardiographic asystole in large swine.

## Supporting information

S1 Text ARRIVE checklist. (PDF)

S2 Text Protocol and analysis plan. (DOCX)

S1 Data Supplementary data file. (XLSX)

## Abbreviations

BP: blood pressure
CO: cardiac output
CPDA-1: standard citrate
CVP: central venous pressure
ECLS: extracorporeal life support
ETCO_2_: end-tidal carbon dioxide
FWB: fresh whole blood
IACUC: institutional animal care and use committee
IV: intravenous
LOST: low-output state in trauma
LR: lactated Ringer’s
NCTH: noncompressible torso hemorrhage
NIRS: near-infrared spectroscopy
NOST: no-output state in trauma
PEA: pulseless electrical activity
REBOA: Resuscitative Endovascular Balloon Occlusion of the Aorta
SAAP: Selective Aortic Arch Perfusion
SAAP-FWB: Selective Aortic Arch perfusion with 1,600 ml of intra-aortic oxygenated fresh whole blood
SAAP-LR: Selective Aortic Arch Perfusion with 1,600 ml of intra-aortic oxygenated lactated Ringer’s solution
SBP: systolic blood pressure
SV: stroke volume
SVR: systemic vascular resistance
TCA: traumatic cardiac arrest

## References

[1] World Health Organization. Injuries and Violence: the Facts. 2014. http://www.who.int/violence_injury_prevention/media/news/2015/Injury_violence_facts_2014/en/

[2] Office for National Statistics. Deaths Registered in England and Wales: 2014. https://www.ons.gov.uk/peoplepopulationandcommunity/birthsdeathsandmarriages/deaths/bulletins/deathsregistrationsummarytables/2015-07-15

[3] National Center for Health Statistics (U.S.). Catalog of Publications… from the Centers for Disease Control and Prevention, National Center for Health Statistics. 1998.

[4] Kauvar DS, Lefering R, Wade CE. Impact of hemorrhage on trauma outcome: an overview of epidemiology, clinical presentations, and therapeutic considerations. The Journal of Trauma: Injury, Infection, and Critical Care. 2006 6;60(6 Suppl):S3–11. 10.1097/01.ta.0000199961.02677.19 16763478

[5] Chiara O, Scott JD, Cimbanassi S, Marini A, Zoia R, Rodriguez A, et al Trauma deaths in an Italian urban area: an audit of prehospital and in-hospital trauma care. Injury. Elsevier; 2002 1 9;33(7):553–62. 10.1016/s0020-1383(02)00123-7 12208056

[6] Tien HC, Spencer F, Tremblay LN, Rizoli SB, Brenneman FD. Preventable deaths from hemorrhage at a level I Canadian trauma center. The Journal of Trauma: Injury, Infection, and Critical Care. 2007 1;62(1):142–6. 10.1097/01.ta.0000251558.38388.47 17215745

[7] Davis JS, Satahoo SS, Butler FK, Dermer H, Naranjo D, Julien K, et al An analysis of prehospital deaths: Who can we save? J Trauma Acute Care Surg. 2014 8;77(2):213–8. doi: 10.1097/TA.0000000000000292 25058244 10.1097/TA.0000000000000292

[8] Teixeira PGR, Inaba K, Hadjizacharia P, Brown C, Salim A, Rhee P, et al Preventable or potentially preventable mortality at a mature trauma center. J Trauma. 2007 12;63(6):1338–46–discussion1346–7. 18212658 10.1097/TA.0b013e31815078ae

[9] Eastridge BJ, Mabry RL, Seguin P, Cantrell J, Tops T, Uribe P, et al Death on the battlefield (2001–2011): implications for the future of combat casualty care. J Trauma Acute Care Surg. 2012 12;73(6 Suppl 5):S431–7. doi: 10.1097/TA.0b013e3182755dcc 23192066 10.1097/TA.0b013e3182755dcc

[10] Morrison JJ, Stannard A, Rasmussen TE, Jansen JO, Tai NRM, Midwinter MJ. Injury pattern and mortality of noncompressible torso hemorrhage in UK combat casualties. J Trauma Acute Care Surg. 2013 8;75(2 Suppl 2):S263–8. doi: 10.1097/TA.0b013e318299da0a 23883918 10.1097/TA.0b013e318299da0a

[11] HUGHES CW. Use of an intra-aortic balloon catheter tamponade for controlling intra-abdominal hemorrhage in man. Surgery. 1954 7;36(1):65–8. 13178946

[12] Gupta BK, Khaneja SC, Flores L, Eastlick L, Longmore W, Shaftan GW. The role of intra-aortic balloon occlusion in penetrating abdominal trauma. The Journal of Trauma: Injury, Infection, and Critical Care. 1989 6;29(6):861–5. 10.1097/00005373-198906000-00026 2661845

[13] Stannard A, Eliason JL, Rasmussen TE. Resuscitative endovascular balloon occlusion of the aorta (REBOA) as an adjunct for hemorrhagic shock. J Trauma. 2011 12;71(6):1869–72. doi: 10.1097/TA.0b013e31823fe90c 22182896 10.1097/TA.0b013e31823fe90c

[14] Avaro J-P, Mardelle V, Roch A, Gil C, de Biasi C, Oliver M, et al Forty-Minute Endovascular Aortic Occlusion Increases Survival in an Experimental Model of Uncontrolled Hemorrhagic Shock Caused by Abdominal Trauma. The Journal of Trauma: Injury, Infection, and Critical Care. 2011 9;71(3):720–6. 10.1097/TA.0b013e318221a94a 21909002

[15] Morrison JJ, Percival TJ, Markov NP, Villamaria C, Scott DJ, Saches KA, et al Aortic balloon occlusion is effective in controlling pelvic hemorrhage. J Surg Res. Elsevier; 2012 10;177(2):341–7. 10.1016/j.jss.2012.04.035 22591921

[16] Morrison JJ, Ross JD, Houston R, Watson JDB, Sokol KK, Rasmussen TE. Use of resuscitative endovascular balloon occlusion of the aorta in a highly lethal model of noncompressible torso hemorrhage. Shock. 2014 2;41(2):130–7. doi: 10.1097/SHK.0000000000000085 24430492 10.1097/SHK.0000000000000085

[17] Martinelli T, Thony F, Decléty P, Sengel C, Broux C, Tonetti J, et al Intra-aortic balloon occlusion to salvage patients with life-threatening hemorrhagic shocks from pelvic fractures. J Trauma. 2010 4;68(4):942–8. doi: 10.1097/TA.0b013e3181c40579 20173661 10.1097/TA.0b013e3181c40579

[18] Brenner ML, Moore LJ, DuBose JJ, Tyson GH, McNutt MK, Albarado RP, et al A clinical series of resuscitative endovascular balloon occlusion of the aorta for hemorrhage control and resuscitation. Journal of Trauma and Acute Care Surgery. 2013 9;75(3):506–11. doi: 10.1097/TA.0b013e31829e5416 24089121 10.1097/TA.0b013e31829e5416

[19] Sadek S, Lockey DJ, Lendrum RA, Perkins Z, Price J, Davies GE. Resuscitative endovascular balloon occlusion of the aorta (REBOA) in the prehospital setting: An additional resuscitation option for uncontrolled catastrophic haemorrhage. Resuscitation. 2016 7 1. 10.1016/j.resuscitation.2016.06.029 27377669

[20] Norii T, Crandall C, Terasaka Y. Survival of severe blunt trauma patients treated with resuscitative endovascular balloon occlusion of the aorta compared with propensity score-adjusted untreated patients. Journal of Trauma and Acute Care Surgery. 2015;:1. 10.1097/TA.0000000000000578 25742248

[21] Manning JE, Murphy CA, Hertz CM, Perretta SG, Mueller RA, Norfleet EA. Selective aortic arch perfusion during cardiac arrest: a new resuscitation technique. Ann Emerg Med. 1992 9;21(9):1058–65. 1514716 10.1016/s0196-0644(05)80645-6

[22] Manning JE, Batson DN, Payne FB, Adam N, Murphy CA, Perretta SG, et al Selective aortic arch perfusion during cardiac arrest: enhanced resuscitation using oxygenated perflubron emulsion, with and without aortic arch epinephrine. Ann Emerg Med. 1997 5;29(5):580–7. 9140240 10.1016/s0196-0644(97)70244-0

[23] Manning JE, Katz LM, Pearce LB, Batson DN, McCurdy SL, Gawryl MS, et al Selective aortic arch perfusion with hemoglobin-based oxygen carrier-201 for resuscitation from exsanguinating cardiac arrest in swine. Crit Care Med. 2001 11;29(11):2067–74. 11700396 10.1097/00003246-200111000-00005

[24] Ross JD, Burns CJ, Sagini EM, Zarzabal L-A, Morrison JJ. A laparoscopic swine model of noncompressible torso hemorrhage. J Trauma Acute Care Surg. 2014 9;77(3 Suppl 2):S77–82. doi: 10.1097/TA.0000000000000385 25159366 10.1097/TA.0000000000000385

[25] Morrison JJ, Ross JD, Rasmussen TE, Midwinter MJ, Jansen JO. Resuscitative endovascular balloon occlusion of the aorta: A gap analysis of severely injured UK combat casualties. Shock. 2014 5;41(5):388–93. doi: 10.1097/SHK.0000000000000136 25133599 10.1097/SHK.0000000000000136

[26] Gräsner J-T, Wnent J, Seewald S, Meybohm P, Fischer M, Paffrath T, et al Cardiopulmonary resuscitation traumatic cardiac arrest—there are survivors. An analysis of two national emergency registries. Crit Care. BioMed Central Ltd; 2011;15(6):R276. 10.1186/cc10558 PMC3388703 22108048

[27] Deakin CD, Low JL. Accuracy of the advanced trauma life support guidelines for predicting systolic blood pressure using carotid, femoral, and radial pulses: observational study. BMJ. 2000 9 16;321(7262):673–4. 10987771 10.1136/bmj.321.7262.673 PMC27481

[28] Lockey D, Crewdson K, Davies G. Traumatic cardiac arrest: who are the survivors? Ann Emerg Med. 2006 9;48(3):240–4. doi: 10.1016/j.annemergmed.2006.03.015 16934644 10.1016/j.annemergmed.2006.03.015

[29] Rosemurgy AS, Morris, Olson SM, Hurst JM, Albrink MH. PREHOSPITAL TRAUMATIC CARDIAC ARREST: THE COST OF FUTILITY. Journal of Trauma and Acute Care Surgery. 1993 9 1;35(3):468. 8371308

[30] Barnard E, Yates D, Edwards A, Fragoso-Iñiguez M, Jenks T, Smith JE. Epidemiology and aetiology of traumatic cardiac arrest in England and Wales—A retrospective database analysis. Resuscitation. 2017;110:90–94. doi: 10.1016/j.resuscitation.2016.11.001 27855275 10.1016/j.resuscitation.2016.11.001

[31] Duchateau F-X, Hamada S, Raux M, Gay M, Mantz J, Paugam Burtz C, et al Long-term prognosis after out-of-hospital resuscitation of cardiac arrest in trauma patients: prehospital trauma-associated cardiac arrest. Emerg Med J. 2017 1;34(1):34–8. doi: 10.1136/emermed-2014-204596 27797869 10.1136/emermed-2014-204596

[32] Lockey DJ, Lyon RM, Davies GE. Development of a simple algorithm to guide the effective management of traumatic cardiac arrest. Resuscitation. Elsevier; 2013 6;84(6):738–42. 10.1016/j.resuscitation.2012.12.003 23228555

[33] Sherren PB, Reid C, Habig K, Burns BJ. Algorithm for the resuscitation of traumatic cardiac arrest patients in a physician-staffed helicopter emergency medical service. Crit Care. BioMed Central Ltd; 2013;17(2):308. 10.1186/cc12504 PMC3672499 23510195

[34] Smith JE, Rickard A, Wise D. Traumatic cardiac arrest. J R Soc Med. 2015 1;108(1):11–6. doi: 10.1177/0141076814560837 25572990 10.1177/0141076814560837 PMC4291327

[35] National Association of EMS Physicians, American College of Surgeons Committee on Trauma. Withholding of resuscitation for adult traumatic cardiopulmonary arrest. Prehosp Emerg Care. 2013 4;17(2):291 doi: 10.3109/10903127.2012.755586 23327549 10.3109/10903127.2012.755586

[36] Kinnear-Mellor R, Newton K, Woolley T, Rickard R. Predictive utility of cardiac ultrasound in traumatic cardiac arrest in a combat casualty. J R Army Med Corps. British Medical Journal Publishing Group; 2015 3 3;:jramc–2014–000358. 10.1136/jramc-2014-000358 25736444

[37] Markov NP, Percival TJ, Morrison JJ, Ross JD, Scott DJ, Spencer JR, et al Physiologic tolerance of descending thoracic aortic balloon occlusion in a swine model of hemorrhagic shock. Surgery. Elsevier; 2013 6;153(6):848–56. 10.1016/j.surg.2012.12.001 23453327

[38] White JM, Cannon JW, Stannard A, Markov NP, Spencer JR, Rasmussen TE. Endovascular balloon occlusion of the aorta is superior to resuscitative thoracotomy with aortic clamping in a porcine model of hemorrhagic shock. Surgery. Elsevier; 2011 9;150(3):400–9. 10.1016/j.surg.2011.06.010 21878225

[39] Barnard EBG, Morrison JJ, Madureira RM, Lendrum R, Fragoso-Iñiguez M, Edwards A, et al Resuscitative endovascular balloon occlusion of the aorta (REBOA): a population based gap analysis of trauma patients in England and Wales. Emerg Med J. 2015 12;32(12):926–32. doi: 10.1136/emermed-2015-205217 26598631 10.1136/emermed-2015-205217 PMC4717355

[40] Sadek S, Lockey DJ, Lendrum RA, Perkins Z, Price J, Davies GE. Resuscitative endovascular balloon occlusion of the aorta (REBOA) in the prehospital setting: An additional resuscitation option for uncontrolled catastrophic haemorrhage. Resuscitation 2016, 10;107:135–8. doi: 10.1016/j.resuscitation.2016.06.029 27377669 10.1016/j.resuscitation.2016.06.029

[41] Lamhaut L, Jouffroy R, Soldan M, Phillipe P, Deluze T, Jaffry M, et al Safety and feasibility of prehospital extra corporeal life support implementation by non-surgeons for out-of-hospital refractory cardiac arrest. Resuscitation. Elsevier; 2013 11;84(11):1525–9. 10.1016/j.resuscitation.2013.06.003 23827888

