# Supplementary material for: A comparison of Selective Aortic Arch Perfusion and Resuscitative Endovascular Balloon Occlusion of the Aorta for the management of hemorrhage-induced traumatic cardiac arrest: A translational model in large swine
Source: PLoS Med. 2017 Jul 25;14(7):e1002349. doi: 10.1371/journal.pmed.1002349 (PMC5526509; doi:10.1371/journal.pmed.1002349)
Supplement: S2 Text — (DOCX) [file pmed.1002349.s002.docx]

**Barnard EBG, et al. Analysis Plan for ‘A comparison of Selective Aortic Arch Perfusion and Resuscitative Endovascular Balloon Occlusion of the Aorta for the management of hemorrhage-induced traumatic cardiac arrest: A translational model in large swine’**

**STUDY DESIGN**

This translational laboratory study in large swine received Institutional Animal Care and Use Committee (IACUC) approval on 9^th^ April 2014. Protocol development and training occurred between 22^nd^ July 2014 and 3^rd^ December 2014, after which a final IACUC amendment was approved for the experimental groups. Experimental protocols commenced on 4^th^ December 2014, and were completed on 16^th^ June 2015.

**OBJECTIVES**

The objective of this translational laboratory study is to evaluate the novel resuscitation technique of Selective Aortic Arch Perfusion (SAAP) for the management of hemorrhage-induced traumatic cardiac arrest (TCA).

**HYPOTHESES**

In a large swine model of hemorrhage-induced TCA:

H1. SAAP with intra-aortic oxygenated fresh whole blood will infer a pre-hospital and short-term survival advantage compared to SAAP with intra-aortic oxygenated lactated Ringer’s solution.

H2. SAAP with intra-aortic oxygenated fresh whole blood will infer a pre-hospital and short-term survival advantage compared to Zone 1 Resuscitative Endovascular Balloon Occlusion of the Aorta (REBOA) with high-volume intravenous fresh whole blood.

**STUDY SETTING**

The protocol was performed in a laboratory facility accredited by the American Association for the Accreditation of Laboratory Animal Care.

**STUDY INCLUSION CRITERIA**

- Hematocrit 28-44%
- Platelet count >200,000/mm^3^
- Prothrombin time <12 seconds
- Partial thromboplastin time <24 seconds
- Fibrinogen >100mg/dL
- Animal weight 70-90kg

**STUDY EXCUSION CRITERIA**

- Unexpected death due to technical failure, or as a complication of anesthesia
- Significant blood loss secondary to surgical complication

**SAMPLE SIZE**

SAAP is a novel technique, and in the absence of prior experimental data to estimate effect, the sample size was calculated to be capable of demonstrating a 40% delta in survival (primary outcome); n=12 animals per experimental group.

**STUDY OUTCOMES**

Primary Outcome

- Survival (defined as a systolic blood pressure ≥50mmHg) 60-minutes after the experimental intervention.

Secondary Outcomes:

- Return of spontaneous circulation (ROSC - defined as a systolic blood pressure ≥50mmHg ten minutes after the start of the experimental intervention).
- In animals with a ROSC, the following physiological parameters will be compared at the end of the 60-minute pre-hospital observation period: heart rate, aortic pressure, right atrial pressure, systolic blood pressure, carotid artery flow, calculated coronary artery pressure, calculated pulse pressure, cardiac output, core body temperature, end-tidal carbon dioxide, and near-infrared spectroscopy above and below the diaphragm.
- In animals with a ROSC, the following blood samples will be compared at the end of the 60-minute pre-hospital observation period: arterial blood gas (pH, pO2, pCO2, base excess, potassium, calcium, lactate), complete blood count (hemoglobin, hematocrit, white cell count, platelets), clotting function (prothrombin time, partial thromboplastin time, fibrinogen), and thromboelastography (R, K, alpha-angle, maximum amplitude, lysis-30).

**STATISTICAL ANALYSIS PLAN**

All data will be analyzed and graphically reproduced in GraphPad Prism, La Jolla, CA, USA (version 6.0h). Baseline characteristics will be compared between groups by one-way analysis of variance (ANOVA). Within-group comparisons will be made using Student’s t-test (two-tailed). Statistical analysis of the primary outcome (60-minute survival) will be with a Log-rank (Mantel-Cox) analysis. Analysis of ROSC percentage will be with a Fisher’s exact test. Statistical significance is pre-defined as p<0.05.

As requested by the IACUC an interim analysis is planned at n=6 – the randomization schedule will accommodate this.

**MISSING DATA**

Every effort will be made to record all baseline characteristic, primary, and secondary outcome data (aided by daily laboratory briefs, use of checklists, the use of pre-defined data collection paper forms and spreadsheets, and data backup). In the event of missing data points their relevance will be discussed with the PI, with the default of requesting additional animals to be added to the schedule following IACUC authorizations.
